# Racial biases, facial trustworthiness, and resting heart rate variability: unravelling complexities in pain recognition

**DOI:** 10.1186/s41235-024-00588-0

**Published:** 2024-10-08

**Authors:** Ilenia Ceccarelli, Arianna Bagnis, Cristina Ottaviani, Julian F. Thayer, Katia Mattarozzi

**Affiliations:** 1 Department of Medical and Surgical Sciences, University of Bologna, Via Massarenti 9, Pad. 21, Bologna, Italy; 2 Department of Psychology, Sapienza University of Rome, Rome, Italy; 3 IRCCS Santa Lucia Foundation, Rome, Italy; 4 Department of Psychological Science, University of California, Irvine, Irvine, CA USA

**Keywords:** Appearance-based trustworthiness, Facial appearance, Heart rate variability, Healthcare, Pain recognition

## Abstract

The study explores whether racial identity and appearance-based trustworthiness judgments can affect recognition of pain in medical students differing in levels of resting heart rate variability (HRV), a measure of parasympathetic control of the heart. After undergoing HRV assessment, 68 medical students (37 females) participated in a dynamic pain recognition task, viewing video clips of White and Black faces, which differed in perceived trustworthiness based on facial appearance, transitioning from neutral to intense pain expressions. Response time, pain intensity attribution and treatment recommendations were analyzed. Pain was recognized slower and estimated as less intense in Black compared to White faces, leading to a lower likelihood of recommending therapy. Pain recognition was faster for untrustworthy-looking White faces compared to trustworthy ones, while perceived trustworthiness had a minimal impact on the speed of pain recognition in Black faces. However, untrustworthy-looking faces were estimated to express more pain, particularly for Black faces. Notably, these biases were more pronounced in individuals with low, rather than high, resting HRV. Considering that therapeutic decisions mirrored pain intensity attribution, it would be important to increase awareness of these biases during medical training in order to promote equity in future pain assessment and treatment.

**Supplementary Information:**

The online version contains supplementary material available at 10.1186/s41235-024-00588-0.

## Introduction

Recognizing pain is an important skill that humans have developed to ensure survival (Schiefenhövel, 1995; Williams, 2002). Among the different social cues people may use to recognize pain in others (Fordyce, 1976), facial expression is one of the most prominent and reliable non-verbal pain indicators (Craig et al., 2011).

Painful facial expression is characterized by distinct facial features (i.e., tightening of the eyelids, lowering the brow regions and nose wrinkling/upper lip raising) that makes it discernible from other facial expressions (Kunz et al., 2019; Prkachin & Solomon, 2008). Importantly, the facial expression of pain shares important characteristics with emotional displays, especially in terms of specificity and generalizability (Chen et al., 2018; Cordaro et al., 2018; Mende-Siedlecki et al., 2021) and its recognition appears to be robust and automatic (Simon et al., 2008). Research on social cognition has repeatedly demonstrated that social categories derived from the invariant features of the face, such as racial identity, interact with variant facial expressions of emotion to influence their recognition (Bagnis et al., 2019; Craig & Lipp, 2017; Herlitz & Lovén, 2013; Hewstone et al., 2002; Macrae & Bodenhausen, 2000; Mason et al., 2006; Wacker et al., 2017). For example, White perceivers appear to detect sadness less readily in Black compared to White faces (Mende-Siedlecki et al., 2021). Moreover, White people tend to overperceive anger (Hugenberg, 2005; Hugenberg & Bodenhausen, 2003; Shapiro et al., 2009) and underperceive happiness (Friesen et al., 2019; Hugenberg, 2005) when Black targets are displayed.

Importantly, this racial bias intensifies when painful faces are presented (Mende-Siedlecki et al., 2021) with studies conducted in clinical settings alarmingly indicating that pain experienced by Black compared to White patients is often underestimated and underdiagnosed (Anderson et al., 2009; Green et al., 2003; Staton et al., 2007). Moreover, Black patients are less likely to be prescribed opioids and pain killers in general; and when they receive them, such medications are usually prescribed at lower doses (Becker et al., 2011; Chen et al., 2005; Olsen et al., 2006; Tamayo-Sarver et al., 2003). Overall, a growing body of laboratory evidence demonstrates that White participants perceive pain more readily in White compared to Black faces (Lin et al., 2020; Mende-Siedlecki et al., 2019, 2022). This bias persists even when Black and White facial stimuli are equated in terms of contrast, luminance, hue, structure, and expressions; and it emerges also under minimal presentation conditions (i.e., 33 ms) (Mende-Siedlecki et al., 2022), suggesting the involvement of a more featural, rather than configural, face processing mechanism (Lin et al., 2020).

Independent studies on face perception suggest that human faces convey information about personality traits based solely on their invariant physical features (Todorov et al., 2015). These appearance-based attributions are rapid and effortless (Montepare & Dobish, 2003; Todorov et al., 2015), and encompass various social characteristics such as dominance, competence, and trustworthiness (Rule et al., 2012). Facial appearance-based inferences can influence healthcare providers' caring inclination (Mattarozzi et al., 2017). For instance, patients' attractiveness has been linked to doctors' judgments about pain (Hadjistavropoulos et al., 1990), while perceived trustworthiness from facial appearance has been associated with a higher likelihood of receiving priority treatment in emergency units (Bagnis et al., 2020a). Studies particularly evidenced that perceived trustworthiness may affect doctors’ judgments about the authenticity or intentions of patients (Schäfer et al., 2016). Furthermore, inferences of trustworthiness from facial appearance can impact emotion recognition, with untrustworthy-looking faces leading to reduced accuracy and speed in recognizing emotion compared to trustworthy-looking faces (Colonnello et al., 2019). It is important to mention that perceived trustworthiness is the social dimension that best approximates the intrinsic valence of a face, correlating with both positive and negative judgments (Todorov et al., 2021).

In this regard, resting vagally—mediated Heart Rate Variability (HRV)—a measure of parasympathetic control of the heart*—*has been consistently considered a reliable biomarker of social engagement (Miller, 2018; Porges, 2011), prosociality (Beffara et al., 2016; Kogan et al., 2014; Miller et al., 2017), emotion regulation (Eisenberg et al., 1996; Geisler et al., 2010, 2013; Lischke et al., 2019; Williams et al., 2015), theory of mind (Zammuto et al., 2021), and emotion recognition (Chaves et al., 2021; Quintana et al., 2012). Moreover, this physiological index has recently emerged as a plausible predictor of individual differences in inferring trustworthiness from facial appearance (Bagnis et al., 2023), with research outlining a positive correlation between higher resting HRV and an increased tendency to perceive others as more trustworthy. Drawing from the foundational principles of the Neurovisceral Integration Model (Thayer and Lane, 2009), and given that pain is an unpleasant emotional experience by definition (Raja et al., 2020), the above-mentioned findings underscore the role of HRV as a sensitive marker of an individual's ability to respond to and recognize social cues, such as inference of trustworthiness from facial appearance, and emotional expressions, including other’s facial expression such as pain. This suggests a possible interactive effect of resting HRV and perceived trustworthiness on accurately recognizing painful expressions. This effect is vital to consider when investigating racial biases in pain recognition, as it may contribute to inequalities in the treatment of Black and White patients.

**Fig. 1:**
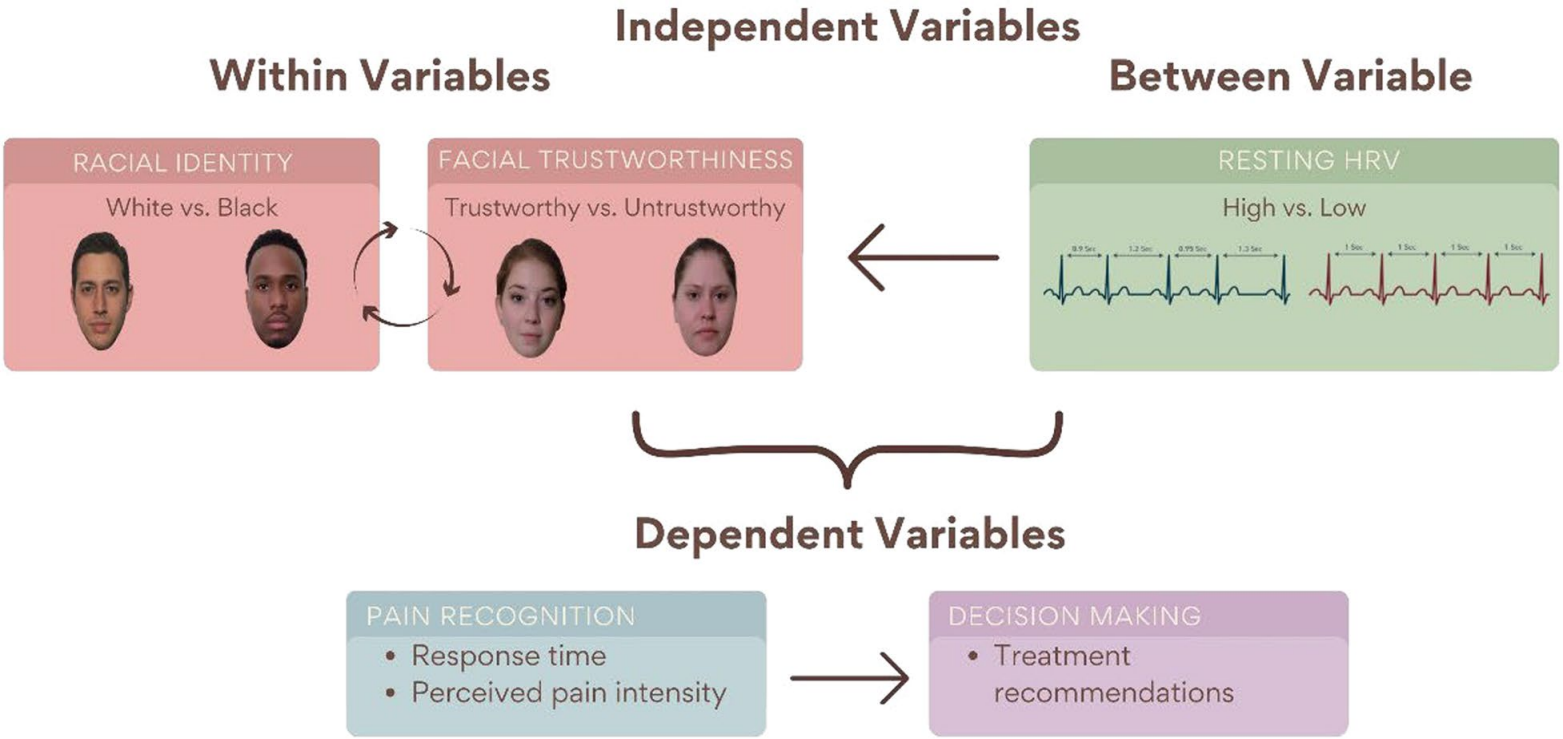
Schematic representation of the main variables involved in the experimental design. *Note* HRV = Heart Rate Variability

The present study has the aim to deepen the current knowledge on the interplay between racial bias, inference of trustworthiness from facial appearance, and individual differences in resting HRV in decoding pain in others. The current investigation specifically examines the effects that facial trustworthiness and racial identity have on pain recognition in individuals with high versus low levels of HRV, used as a proxy of their biological predisposition to recognize emotional states in others (Fig. 1). Due to the relevance of these processes in the healthcare field and the consequences they may generate in terms of provided treatments, we recruited potential future doctors, namely a sample of medical students.

In line with the reviewed evidence, we hypothesize that facial characteristics and racial biases will significantly impact pain recognition. Specifically, we expect that Black and untrustworthy-looking faces will lead to impaired pain recognition, which can ultimately influence therapeutic decisions. We further hypothesize that these effects will be particularly pronounced in medical students characterized by low resting HRV. This prediction is grounded in the understanding that low HRV is associated with diminished social engagement and emotion regulation capabilities (Geisler et al., 2013; Williams et al., 2015), which could impair the accurate recognition of pain expressions.

To enhance the ecological validity of our investigation and ensure that our findings are relevant to real-world scenarios, we employed a dynamic pain recognition task. This task involved presenting participants with video clips depicting facial expressions that morph from neutral to full-intensity pain expressions, simulating the natural variability and complexity of emotional expressions as they occur in real life. This methodological approach allows us to capture the nuances of pain recognition more effectively than static images would, providing a more accurate measure of how medical students perceive and respond to pain expressions in a dynamic context.

## Materials and methods

### Participants

The experimental procedure was approved by the Institutional Review Board of the University of Bologna, Italy. All participants signed an informed consent prior the study, and they were fully debriefed about the research question at the end of their participation.

Sixty-eight medical students (37 females, 31 males, age range: 19–31 years, *M* = 20.73, SD = 1.90 years) from the University of Bologna were recruited. Sample size was established through an a-priori calculation using G*Power software (Faul et al., 2007), which indicated that assuming a small effect size (*f* = 0.20) and a correlation of 0.50 among repeated measures, a minimum of 56 participants would be needed to achieve a statistical power of 0.95 for alpha = 0.05.

### Procedure

The study was composed of three different phases. First, participants underwent resting HRV assessment. Second, they were asked to perform the pain recognition task, in which both White and Black faces, differing in trustworthiness inferred from facial appearance, were presented. Specifically, for each of the dynamic painful stimuli, they were asked to (1) stop the video when a painful expression was recognized, (2) indicate the perceived pain intensity, and (3) report the probability with which they would administer a therapy to the displayed putative patient. Lastly, the Implicit Association Test (IAT) (Greenwald et al., 1998) was administered as a measure of implicit racial bias.

### Heart rate variability assessment

Participants were instructed not to engage in strenuous exercise on the day of the study and to abstain from alcohol, caffeine, and nicotine within two hours prior to their participation. Upon arrival at the laboratory, participants were invited in a room where they could seat comfortably. After the electrocardiography (ECG) electrodes were placed on their chest, participants were instructed to sit still and breathe spontaneously, and they were left alone in the room for 6 minutes. ECG was assessed according to the “vanilla baseline” procedure, during which they looked at a neutrally valenced (i.e., gardening) magazine alone in the room (Jennings et al., 1992).

Heart rate was recorded as beat- to- beat intervals in ms with the Bodyguard 2 (Firstbeat Technologies Ltd.; Parak & Korhonen, 2013). HRV was derived by computing the root mean square of successive beat-to-beat interval differences (rMSSD), which reflects vagal regulation of HR with reduced susceptibility to respiratory influences (Laborde et al., 2017; Penttilä et al., 2001). Analyses were performed using Kubios HRV software (Tarvainen et al., 2014), which uses an advanced detrending method based on the formulation of smoothing priorities (Tarvainen et al., 2002). Artifacts and ectopic beats were corrected using Kubios HRV software by two different methods: (1) A threshold-based correction, which is based on the comparison of each RR interval value to a local average interval; (2) an automatic correction, where artifacts are detected from a time series consisting of the differences between successive RR intervals.

### Pain recognition task

After ECG recording, participants were asked to sit in front of the computer screen (monitor dimension: 22 × 49.2 × 37.6 cm; visual angle: 178°) where the task would be administered. They received oral and written instructions, and two practice trials were given to become familiar with the task, which was composed of 16 trials in total. Each trial was preceded by a central fixation cross. The video clips presentation order was pseudorandomized controlling for trustworthiness and racial identity. The total duration of the task was ~ 20 min. Participants were instructed to view each video and press the keyboard spacebar as soon as they decoded the expression depicted in the video clip as a painful expression. Immediately after stopping the video, they were asked to report the intensity of the displayed pain, as well as the probability with which they would give the therapy to the target if they were the doctor in charge to treat them. Participants made these latter two responses based on the still image at the point when they stopped the video—i.e., once they saw pain on the face. The video did not continue to play to the end.

This methodological choice is advantageous as it ensures that the intensity and treatment judgments are made based on the exact moment that participants perceive pain. This design effectively equates the phenomenological quality of the stimulus across different conditions (e.g., race or trustworthiness) at the "tipping point" for pain detection. Doing so allowed us to control for potential biases that could arise from different onsets of pain perception in the videos. Specifically, this approach reduces the influence of any inherent biases in pain detection timing on subsequent judgments. Even if participants perceived pain earlier in one condition (e.g., White or trustworthy faces) compared to another (e.g., Black or untrustworthy faces), this bias is carried over to intensity and treatment ratings, regardless the stopped frame. Thus, our method helps isolate and accurately measure the impact of perceived racial identity and trustworthiness on pain intensity ratings and treatment recommendations, without conflating these effects with differences in the timing of pain detection.

Response time in milliseconds (ms), perceived pain intensity and probability of giving the therapy on Likert scales (0 = not at all intense to 10 = extremely intense; 0 = not at all likely to 10 = extremely likely, respectively) were recorded. For stimulus presentation and response data collection, E-Prime 2 software (Psychology software tools, Pittsburgh, USA) was used.

A total of 16 video clips (10s each, 25 frames/s) were used as stimuli. Each video showed a neutral facial expression gradually and continuously changing into a full-intensity painful facial expression. To build the stimuli, 72 frontal color photographs of the faces of 8 Black and 8 White actors were used. We selected the most trustworthy-looking and untrustworthy- looking faces from the Delaware Pain Database (Mende-Siedlecki et al., 2020), ensuring they did not differ in this variable across racial identity and sex (Table 1). The stimuli were controlled for perceived pain intensity as well. Specifically, the mean perceived pain intensity for trustworthy faces was 3.86 (SD = 0.66), while for untrustworthy faces it was 4.21 (SD = 0.71) (*t* = − 1.04, *p* = 0.31). The selection was also controlled across race in terms of perceived pain intensity. The mean perceived pain intensity for White faces was 4.35 (SD = 0.74), while for Black faces it was 3.71 (SD = 0.49), (*t* = 2.04, *p* = 0.07). This indicates that any variation in perceived pain intensity was minimal and not likely to influence the results. Finally, stimuli were also controlled for any potential latent pain content in neutral expressions, by selecting faces with minimal or no perceived pain in their neutral state as recorded in the Delaware Pain Database.

**Table 1:** Descriptive (M ± SD) and comparison statistics of selected stimuli

	Racial identity			
	White	Black	*t*^a^	*p*
Trustworthy-looking faces	4.08 ± 0.19	3.95 ± 0.14	1.06	0.33
Untrustworthy-looking faces	2.76 ± 0.30	2.83 ± 0.38	− .30	0.77

	Sex			
	Female	Male	*t*^a^	*p*
Trustworthy-looking faces	3.95 ± 0.20	4.08 ± 0.11	− 1.05	0.33
Untrustworthy-looking faces	2.94 ± 0.13	2.64 ± 0.23	2.27	0.07

^a^*df* = 6

For each actor, we chose images representing both a neutral and a painful facial expression. Moreover, two additional neutral/painful faces of two other actors were used to create video clips for the practice trials.

Each image was manipulated to remove extraneous attributes (e.g., hair) and subjected to morphing by means of FantaMorph© software (Abrosoft, http://www.fantamorph.com/index.html). For each actor, morph sequences were created with increasing painful intensity, where the neutral and painful faces represented, respectively, the first and last frames. Lastly, a video clip was composed for each of the selected actors.

### Implicit Association Test

Participants lastly performed the IAT to measure implicit racial bias (Greenwald et al., 1998). The task was composed of 7 blocks, and it measured the strength of automatic associations between two groups (White and Black) and two concepts (positive or negative). For each trial, participants were required to pair Black and White faces with either positive (e.g., happiness) or negative (e.g., agony) words as fast as possible. The strength of associations was measured by calculating the differences in terms of response latencies between Black/positive and White/negative pairings compared to the Black/negative and White/positive pairings. D values greater than zero indicates an implicit preference for White versus Black people (Greenwald & Banaji, 1995; Greenwald et al., 1998; Lane et al., 2007).

### Statistical analysis

Differences among the two groups in terms of sex distribution, age, and Body Mass Index (BMI) were calculated using *χ*^2^-tests and *t*-tests as appropriate. Sex, age, and BMI were examined as potential covariates as they have been shown to be related to HRV (Antelmi et al., 2004; Koenig & Thayer, 2016; Reardon et al., 1996).

A median split was used to differentiate between participants with low HRV and high HRV (Iacobucci et al., 2015a, 2015b).

A series of mixed 2 × 2 × 2 Analysis of Variance (ANOVA) were performed on pain detection (i.e., the response time in ms needed to recognize a painful expression), pain intensity, and probability of providing the therapy. Facial trustworthiness (trustworthy, untrustworthy) and racial identity (Black, White) were considered as within-subject factors; while, HRV (low, high) was considered as a between—subject factor. Post hoc Bonferroni-corrected comparisons were run.

Effect sizes were calculated using partial eta squared. The alpha level for all analyses was set to *p <* 0.05. All the analyses were run using SPSS version 25.0 (Chicago, IL).

## Results

Both low (23 females; *M* = 36.11, *SD* = 10.84) and high (14 females; *M* = 96.51, *SD* = 45.75) HRV groups were composed of 34 medical students. The two groups differ in terms of sex distribution (*χ*^2^ = 4.80; *p* = 0.03), but no differences were found in terms of age (*t*(66) = − 1.23; *p* = 0.23) and BMI (*t*(66) = − 0.70; *p* = 0.49). Therefore, sex was included in the analyses as a between-subject factor; while, age and BMI were not considered as covariates. Table S1 reports the correlation coefficient and p value demonstrating the orthogonality between the median split variable (rMSSD) and the independent variables, as suggested by Iacobucci et al., (2015a, 2015b). Considering that results did not change when the analyses were repeated with the inclusion of scores on the IAT as a covariate, they will not be reported here. Statistical details for all non-significant results are available in the supplementary file.

### Response time

A main effect of Racial identity on pain detection emerged (*F*(1,64) = 28.80, *p* < 0.001, *η*_*p*_^2^ = 0.31), with medical students being faster in perceiving pain when White (*M* = 4077.20 ms, *SD* = 113.62) compared to Black (*M* = 4432.67 ms, *SD* = 127.67) faces were presented.

**Fig. 2:**
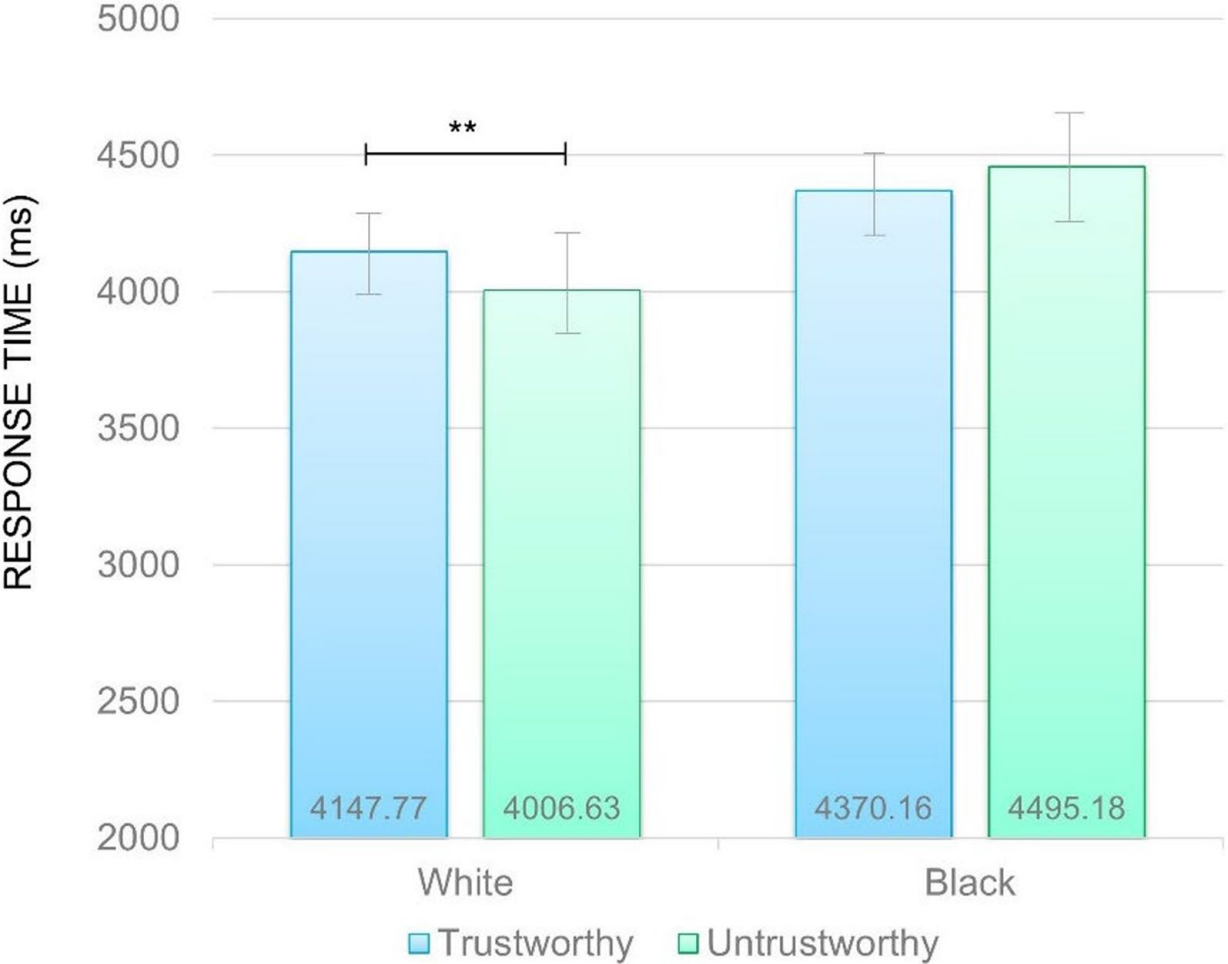
Response time (ms) for the pain recognition on White and Black targets in trustworthy-looking and trustworthy-looking faces. Error bars represent standard error of the mean. *Note* **p < 0.01

As shown in Fig. 2, a statistically significant Racial identity by Trustworthiness interaction was also observed (*F*(1,64) = 5.72, *p* = 0.02, *η*_*p*_^2^ = 0.08). When White faces were administered, slower response times for trustworthy-looking compared to untrustworthy-looking faces emerged (*p* = 0.02), with no differences for Black trustworthy compared to untrustworthy-looking faces.

No other significant main effect or interaction emerged.

### Pain intensity

A main effect of Racial identity on pain intensity was observed, with White faces (*M* = 5.84, *SD* = 0.14) being perceived as characterized by more intense pain compared to Black faces (*M* = 5.15, *SD* = 0.14).

A main effect of Trustworthiness also emerged (*F*(1,64) = 32.81, *p* < .001; *η*_*p*_^2^ = 0.34), with untrustworthy- looking faces (*M* = 5.81, *SD* = 0.13) being evaluated as expressing more pain than trustworthy-looking faces (*M* = 5.17, *SD* = 0.16).

The analysis also yielded a statistically significant Racial identity by Trustworthiness interaction (*F*(1,64) = 14.88, *p* < 0.001, *η*_*p*_^2^ = 0.19), with untrustworthy-looking White faces (*M* = 6.00; *SD* = 0.13) being judged as more in pain than trustworthy-looking White faces (*M* = 5.67, *SD* = 0.17) (*p* < 0.01) and the same but greater difference between untrustworthy-looking (*M* = 5.62, *SD* = 0.15) and trustworthy-looking (*M* = 4.67, *SD* = 0.17) Black faces (*p* < 0.001).

**Fig. 3:**
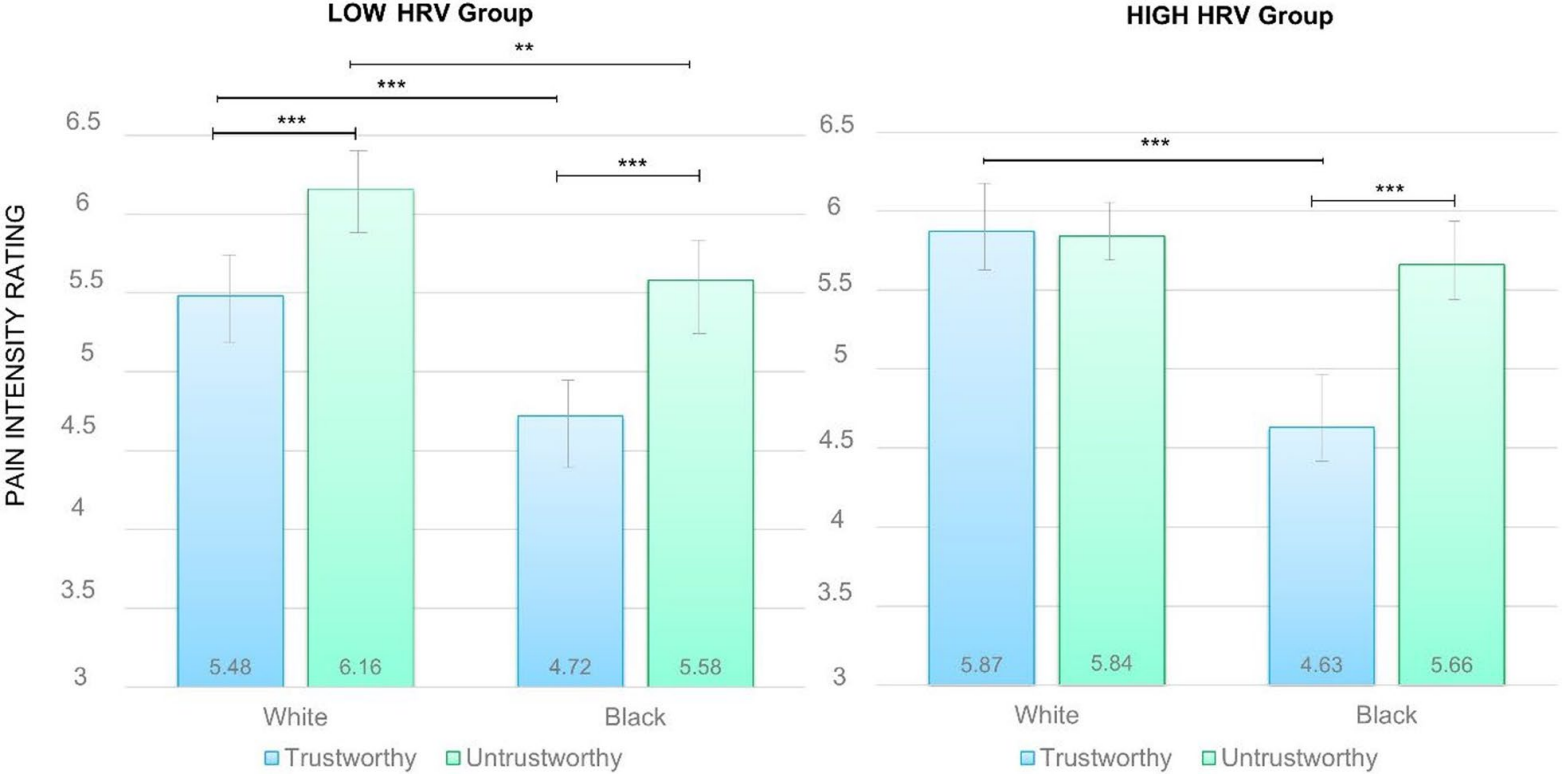
Pain intensity ratings on trustworthy-looking and trustworthy-looking White and Black faces by HRV group. Error bars represent standard error of the mean. *Note* HRV= Heart Rate Variability; **p < 0.01, ***p < 0.001

As shown in Fig. 3, a 3-way statistically significant interaction between Group (low vs high HRV), Racial identity and Trustworthiness was also found (*F*(1,64) = 7.58, *p =* 0.008, *η*_*p*_^2^ = 0.11). Specifically, in the low HRV group, both untrustworthy and trustworthy-looking White faces were rated as being in more pain compared to untrustworthy and trustworthy-looking Black faces, respectively (*p* < 0.001). In the high HRV group, this difference remained significant only for trustworthy-looking White and Black faces (*p* < 0.001), with no differences in the self-evaluation of pain levels in untrustworthy-looking White and Black faces (*p* = 0.30). Additionally, while in the low HRV group untrustworthy-(vs trustworthy-) looking faces were always recognized as being more in pain irrespective of Racial identity (*p* < 0.001), in the high HRV group this was true only for Black faces (*p* < 0.001).

### Treatment recommendations

Main effects of Racial identity (*F*(1,64) = 30.84, *p* < 0.001, *η*_*p*_^2^ = 0.33) and Trustworthiness (*F*(1,64) = 32.32, *p* < 0.001, *η*_*p*_^2^ = 0.34) on treatment recommendations were observed. Specifically, White faces (*M* = 5.25, *SD* = 0.19) were always associated with a higher probability of receiving the therapy compared to Black faces (*M* = 4.68, *SD* = 0.17) (*p* < 0.001) and untrustworthy-looking faces (*M* = 5.31, *SD* = 0.18) were always associated with higher probability of receiving the therapy compared to trustworthy-looking faces (*M* = 4.62, *SD* = 0.20) (*p* < 0.001).

The analysis also yielded a Racial identity by Trustworthiness interaction (*F*(1,64) = 8.76, *p* ≤ 0.004, *η*_*p*_^2^ = 0.12) with untrustworthy-looking White faces (*M* = 5.44, *SD* = 0.19) being more likely to receive therapy compared to trustworthy-looking White faces (*M* = 5.05, *SD* = 0.22) (*p* = 0.009) and an even larger effect when Black faces were considered (untrustworthy-looking Black faces: *M* = 5.17, *SD* = 0.19; trustworthy-looking Black faces: *M* = 4.18, *SD* = 0.20; *p* < 0.001).

Figure 1S depicts the statistically significant Racial identity by Trustworthiness by HRV group interaction that emerged (*F*(1,64) =5.72, *p* = 0.02, *η*_*p*_^2^ = 0.08) that precisely mirrors the results previously described for pain intensity ratings. In the low HRV group, both untrustworthy and trustworthy-looking White faces had higher probability to receive a therapy compared to their Black counterparts (*p* = 0.002). Also, medical students with low resting HRV rated as more likely to administer a therapy to untrustworthy-looking White and Black faces compared to trustworthy-looking White and Black faces, respectively (*p* = 0.002). In the high HRV group, a significant difference (*p* < 0.001) was observed for untrustworthy-looking vs. trustworthy-looking Black faces, with no significant differences (*p* = 0.65) for untrustworthy vs. trustworthy-looking White faces.

A significant Racial identity by HRV group by Sex interaction also emerged, (*F*(1,64) = 5.36, *p* = 0.02, *η*^2^_*p*_ = 0.08). In detail, in the low HRV group, both male (*p* < 0.001) and female (*p* = 0.04) participants were characterized by a higher probability of offering the therapy to White (males: M = 5.24, *SD* = 0.46; females: *M* = 5.32, *SD* = 0.32) compared to Black (males: *M* = 4.27, *SD* = 0.41; females: M = 4.96, *SD* = 0.29) faces. Males in the high HRV group were characterized by the same probability of providing therapy to White (*M* = 4.77, *SD* = 0.34) and Black faces (*M* = 4.45, *SD* = 0.31) (*p* = 0.09); while, high HRV female participants reported a higher probability of administering a therapy to White (*M* = 5.66, *SD* = 0.41) compared to Black, faces (*M* = 5.01, *SD* = 0.37) (*p* = 0.004).

## Discussion

The present study aimed to investigate the interplay between invariant facial features, such as racial identity, and the intrinsic valence associated with appearance-based inferences of trustworthiness, on the recognition of pain expressions. Specifically, we tested whether racial identity and perceived trustworthiness could impact the ability to recognize painful expressions within a cohort of future doctors (i.e., medical students) exhibiting different levels of resting HRV (low versus high).

Results show that, irrespective of perceived trustworthiness, pain was generally recognized less promptly and evaluated as less intense in Black (compared to White) faces by future medical doctors, who also reported a reduced likelihood of offering therapeutic support to Black (compared to White) putative patients. As previously reviewed, the existence of this racial bias in pain perception is well replicated in the literature (Lin et al., 2020; Mende-Siedlecki et al., 2019). To our knowledge, however, this is the first study to examine this in medical students, with a more ecological task (compared to picture viewing) that takes into account crucial features of both the target (i.e., perceived trustworthiness from facial appearance) and the perceiver (i.e., resting HRV and the presence of implicit preferences for White faces).

When White faces were presented, pain was recognized more swiftly in untrustworthy-looking, compared to trustworthy-looking faces. Studies on emotion recognition show that exaggerating faces in the negative or positive direction of the trustworthiness dimension, respectively, increases the attribution of negative (i.e., anger) and positive (i.e., happiness) emotions (Colonnello et al., 2019; Oosterhof & Todorov, 2009). This effect is likely due to the subtle perceptual overlap between emotional facial expressions and the physical attributes characterizing trustworthy and untrustworthy-looking faces (Robinson et al., 2014). Therefore, it is reasonable to hypothesize that when White painful faces are considered, inferences of trustworthiness may modulate pain detection and attribution by priming pain perception in negative valence faces. On the other hand, when Black faces were showcased, the intrinsic valence of the face did not exert a discernible influence on pain detection, pointing toward the presence of a perceptual bias in pain recognition for individuals with Black facial features. The evidence collected in this study suggests that participants engage in feature-based processing when viewing Black faces, which is consistent with findings from previous studies supporting the hypothesis of a perceptual route of racial bias in perceiving pain (Cassidy et al., 2017; Mende-Siedlecki et al., 2019, 2021). Feature-based processing is typically slower than holistic, configural-face processing, which is more commonly observed for same-race faces (Natu & O’Toole, 2011; Walker et al., 2008). This difference in processing may explain the delayed recognition of pain in Black faces compared to White faces in our study. The prevailing literature suggests that emotion recognition, including pain recognition, is supported by configural-face processing (Bombari et al., 2013; Calder & Jansen, 2005; Calder et al., 2000). Therefore, it is reasonable to infer that disruptions in configural processing may compromise the ability to accurately recognize pain expressions. Although our study's design does not allow us to conclusively establish this mechanism, the consistency of our findings with this theoretical framework indicates that feature-based processing could be a contributing factor to the observed racial biases in pain recognition.

Furthermore, while our results did not show a significant impact of IAT scores on pain perception, this does not negate the role of underlying racial biases in face processing. The differential response times and perceived pain intensities between Black and White faces suggest that such biases may be more nuanced and potentially subconscious.

In conclusion, our findings align with the hypothesis that racial biases in pain recognition may stem from differences in how faces are processed. Configural processing, which facilitates emotion recognition, appears to be disrupted when participants view faces of a different race, leading to slower and potentially less accurate pain recognition. Future research should continue to explore this hypothesis and investigate the underlying mechanisms in greater detail.

It is important to acknowledge that factors that were not explicitly assessed in the study, such as interpersonal interactions with other-race people (Lin et al., 2020), and inaccurate biological assumptions regarding Black people (Hoffman et al., 2016) may have contributed to this automatic perceptual racial bias.

When subjective pain evaluation was assessed instead of reaction times, untrustworthy-looking faces were judged as expressing more pain than trustworthy-looking faces, irrespective of their racial category. In the absence of time constraints, a more controlled cognitive process may be involved, enabling judgers to override the automatic perceptual racial bias. Conversely, when assessing response time, heightened readiness may be more vulnerable to automatic biases, leaving no room for conscious correction of the judgement, as highlighted in studies by Todorov (2008) and Willis and Todorov (2006).

At a decisional level, in accordance with the existing literature (Al-Hashimi et al., 2015; Anderson et al., 2009; Lee et al., 2019; Mende-Siedlecki et al., 2019), we observed that results on the probability of providing a therapy overlap those previously observed for pain detection and attribution, suggesting the existence of a coherence between the perceptual bias and medical decisions. Previous studies already suggested that false beliefs about biological differences between blacks and whites influence the way Black people are medically treated (Hoffman et al., 2016); present results further show that those decisions are biased by automatic perceptual processes with alarming consequences for health care.

Notably, present findings provide preliminary evidence that racial and trustworthiness- based biases in pain perception and treatment recommendations may be modulated by individual differences in resting HRV. According to the vast literature on the topic, individuals with lower resting HRV are expected to be characterized by reduced effective prefrontal inhibitory control and therefore by an increased vulnerability to automatic perceptual negative biases (Thayer & Lane, 2000).

Indeed, within the low HRV group, perceived trustworthiness played a discernible role in pain attribution. For both White and Black faces, untrustworthy-looking faces were rated as experiencing more pain than trustworthy-looking ones. In the high HRV group, however, this pattern of results only emerged for Black faces, with no perceived differences between untrustworthy- and trustworthy- looking White faces. This specific effect for White faces is consistent with recent findings highlighting the tendency of individuals with high HRV to perceive others as more trustworthy and less threatening than those with low HRV (Bagnis et al., 2023). Here, it is plausible that familiarity with faces (i.e., White participants looking at White faces) paves the way for this perceptual tendency.

Sex also appeared to influence the extent to which individual differences in HRV modulate the influence of racial bias on medical decisions (Bagnis et al., 2020b; Natu & O’Toole, 2013; Platek & Krill, 2009). This effect may originate from sex-dependent differences in the relationship between amygdalar activation, which has been associated with the other-race face perception (for a meta-analysis, see Bagnis et al., 2020b) and resting HRV (Allen et al., 2015). In fact, while males show a negative relationship between resting HRV and amygdala activation, females are characterized by an opposite pattern of results (Allen et al., 2015). We can speculate that men with higher HRV (and thus with potentially lower amygdala activation) may have perceived Black faces as more approachable, reporting higher probability of prescribing medical treatments to them.

This study is not without limitations. Firstly, the sample was composed entirely of White individuals, limiting the generalizability of the results to members of other racial or ethnic minority groups. Future research should delve into the replication of our findings among individuals with diverse ethnic backgrounds. Additionally, it is essential to acknowledge that the method we utilized, the median split of RMSSD to categorize participants into low and high HRV groups to categorize participants into low and high HRV groups, imposes constraints on internal validity. However, as highlighted by Iacobucci and collaborators, it still yields significant and meaningful information. (Iacobucci et al., 2015a, 2015b). Lastly, our participants were exclusively medical students. While they are expected to possess a greater familiarity with the medical field compared to the general population, and likely exhibit higher motivation to provide care to others due to their educational context, it is important to acknowledge that these results may not necessarily generalize to medical professionals with more extensive experience.

## Conclusions

Racial bias in the health care setting is an incisive global phenomenon in determining disparities between patients in relation to the care they receive. Although findings on racial biases in the healthcare context have been mostly observed in the US, evidence of inequalities in the taking charge of Black and White patients are documented in the European context as well (Fiscella & Sanders, 2016), reporting episodes of verbal abuse, de- prioritization and aggravation of health conditions due to lack of appropriate treatment (Hamed et al., 2020). Overall, the presented data highlights how, in the European context, the interplay between invariant facial features, such as racial identity, and facial appearance-based inferences of trustworthiness, can influence pain detection and evaluation. The results unequivocally affirm the existence of racial disparities in pain perception and treatment recommendations and support the notion that perceptual bias plays a central role in this issue (Lin et al., 2020; Mende-Siedlecki et al., 2019, 2022). The alignment of appearance-based attributions of trustworthiness with a face’s intrinsic valence further underscores the shift from configural face processing to feature-based processing when perceiving pain in Black faces. Lastly, current data adds to the amount of evidence on the role of HRV as an indicator of individuals’ capacity to discern facial expressions and other’s behavioral intentions, suggesting that this increases the vulnerability to racial bias in pain recognition in individuals with lower HRV (Bagnis et al., 2023; Quintana et al., 2012). By shedding light on these processes, it would be possible to inform the development of educational programs to help medical students and physicians to develop strategies aimed to ensure more equitable treatments.

## Supplementary Information

Additional file1

## Data Availability

The datasets used and/or analyzed during the current study are available from the corresponding author on reasonable request.

## Abbreviations

ANOVA: Analysis of variance
BMI: Body mass index
ECG: Electrocardiography
HRV: Heart rate variability
IAT: Implicit Association Test
rMSSD: Root mean square of successive beat-to-beat interval differences
