# Supplementary material for: Racial biases, facial trustworthiness, and resting heart rate variability: unravelling complexities in pain recognition
Source: Cogn Res Princ Implic. 2024 Oct 8;9:69. doi: 10.1186/s41235-024-00588-0 (PMC11461382; doi:10.1186/s41235-024-00588-0)
Supplement: Supplementary file 1 — Additional file1 [file 41235_2024_588_MOESM1_ESM.docx]

**Supplementary Materials**

**Correlations between the median split variable (rMSSD) and the independent variables**

**Table 1S**

Correlations between HRV groups (high and low) and the independent variables

|  | rMSSD | | | |
| --- | --- | --- | --- | --- |
|  | High HRV group  (N= 34) | | Low HRV group  (N= 34) | |
|  | *r* | *p* | *r* | *p* |
| **Response time** |  |  |  |  |
| White trustworthy | 0.03 | 0.85 | 0.12 | 0.51 |
| White untrustworthy | 0.04 | 0.81 | 0.06 | 0.73 |
| Black trustworthy | 0.04 | 0.81 | 0.03 | 0.88 |
| Black untrustworthy | 0.06 | 0.75 | 0.02 | 0.90 |
| **Pain intensity** |  |  |  |  |
| White trustworthy | -0.04 | 0.80 | -0.10 | 0.55 |
| White untrustworthy | -0.08 | 0.67 | -0.22 | 0.20 |
| Black trustworthy | -0.24 | 0.17 | -0.11 | 0.53 |
| Black untrustworthy | 0.03 | 0.86 | -0.08 | 0.67 |
| **Treatment Recommendations** |  |  |  |  |
| White trustworthy | -0.11 | 0.54 | -0.06 | 0.74 |
| White untrustworthy | -0.11 | 0.54 | -0.10 | 0.58 |
| Black trustworthy | -0.24 | 0.17 | -0.03 | 0.86 |
| Black untrustworthy | -0.07 | 0.69 | -0.04 | 0.82 |

**IAT**

We analysed the IAT scores of 64 of the 68 participants. 4 participants were excluded due to technical problems during data collection. Participants showed a moderate pro-white/anti-black bias (M=.73; SD=.37). Table 2S shows the IAT values by HRV group and gender.

**Table 2S.**

Means (± standard deviations) of IAT scores.

|  | HRV group | | | |
| --- | --- | --- | --- | --- |
|  | High (N=33) | Low  (N=31) | *t^1^* | *p* |
| IAT scores | .77 ± .30 | .68 ± .43 | .93 | .36 |
|  | Sex | | | |
|  | Female (N=35) | Male (N=29) | *t^1^* | *p* |
| IAT scores | .69 ± .39 | .77 ± .35 | .83 | .41 |

*Note.* ^1^*df* = 62

**Response Time**

The main effect of Trustworthiness was not statistically significant (*F* (1,64) = .02, *p* = .90, η^2^_p_ = .00). Moreover, neither HRV group nor Sex were significant predictor of response time (main effect of HRV group: *F* (1,64) = .34, *p* = .56, η^2^_p_ = .01; main effect of Sex: *F* (1,64) = .97, *p* = .33, η^2^_p_ = .02). Finally, the effects of HRV group and Sex were not statistically significant even when considered in interaction with the other variables (HRV group x Sex: *F* (1,64) = 1.02, *p* = .32, η^2^_p_ = .02; HRV group x Skin color: *F* (1,64) = .86, *p* = .36, η^2^_p_ = .01; HRV group x Trustworthiness: *F* (1,64) = .94, *p* = .34, η^2^_p_ = .02; HRV group x Skin color x Trustworthiness: *F* (1,64) = 2.76, *p* = .10, η^2^_p_ = .04; HRV group x Skin color x Sex: *F* (1,64) = 1.78, *p* = .19, η^2^_p_ = .03; HRV group x Trustworthiness x Sex: *F* (1,64) = .00, *p* = .99, η^2^_p_ = .00; Sex x Skin color: *F* (1,64) = 2.04, *p* = .16, η^2^_p_ = .03; Sex x Trustworthiness: *F* (1,64) = .65, *p* = .42, η^2^_p_ = .01; Sex x Skin color x Trustworthiness: *F* (1,64) = .61, *p* = .44, η^2^_p_ = .01; Sex x Skin color x Trustworthiness x HRV group: *F* (1,64) = .80, *p* = .37, η^2^_p_ = .01).

**Pain intensity**

HRV group was not a significant predictor of pain intensity neither when considered alone (*F* (1,64) = .01, *p* = .95, η^2^_p_ =.01) nor in interaction with Skin color (*F* (1,64) = .04, *p* = .84, η^2^_p_ = .01), Trustworthiness (*F* (1,64) = 1.48, *p* = .23, η^2^_p_ = .02) and Sex (*F* (1,64) = .41, *p* = .53, η^2^_p_ = .01). Moreover, when the effects of Sex were investigated, no significant findings emerged (main effect of Sex: *F* (1,64) = 2.29, *p* = .14, η^2^_p_ = .04; Sex x Skin color: *F* (1,64) = .15, *p* = .70, η^2^_p_ = .01; Sex x Trustworthiness: *F* (1,64) = .22, *p* = .64, η^2^_p_ = .01; Sex x Skin color x Trustworthiness: *F* (1,64) = .23, *p* = .63, η^2^_p_ = .01; Sex x Skin color x HRV group: *F* (1,64) = 1.57, *p* = .21, η^2^_p_ = .02; Sex x Trustworthiness x HRV group: *F* (1,64) = .01, *p* = .97, η^2^_p_ = .01). The interaction between Skin color, Trustworthiness, HRV group and Sex was not significant as well (*F* (1,64) = .13, *p* = .72, η^2^_p_ = .01).

**Treatment recommendations**

HRV was not a significant predictor of treatment recommendations neither when considered alone (*F* (1,64) =. 01, *p* = .95, η^2^_p_ = .01), nor in interaction with Skin color (*F* (1,64) = .75, *p* = .39, η^2^_p_ = .01), Trustworthiness (*F* (1,64) = .19, *p* = .66, η^2^_p_ = .01) and Sex (*F* (1,64) = .23, *p* = .64, η^2^_p_ = .01). Sex was not a significant predictor as well (*F* (1,64) = 2.49, *p* = .12, η^2^_p_ =.04), and no significant results emerged when the interaction between Sex and Trustworthiness (*F* (1,64) = .48; *p* = .49, η^2^_p_ = .01) and Sex and Skin color (*F* (1,64) = .44, *p* = .51, η^2^_p_ =.01) were analysed. The interactions of Sex, Trustworthiness and Skin color (*F* (1,64) = .68, *p* = .48, η^2^_p_ = .01), and Sex, Trustworthiness and HRV group (*F* (1,64) = .12, *p* = .73, η^2^_p_ = .01), were both not statistically significant. Finally, when the interaction between Skin color, Trustworthiness, Sex and HRV group was considered, no significant results emerged (*F* (1,64) = .35, *p* = .56, η^2^_p_ = .01).

**Figure 1S** Likelihood of treatment recommendations on trustworthy-looking and trustworthy-looking White and Black faces by HRV group. Error bars represent standard error of the mean.


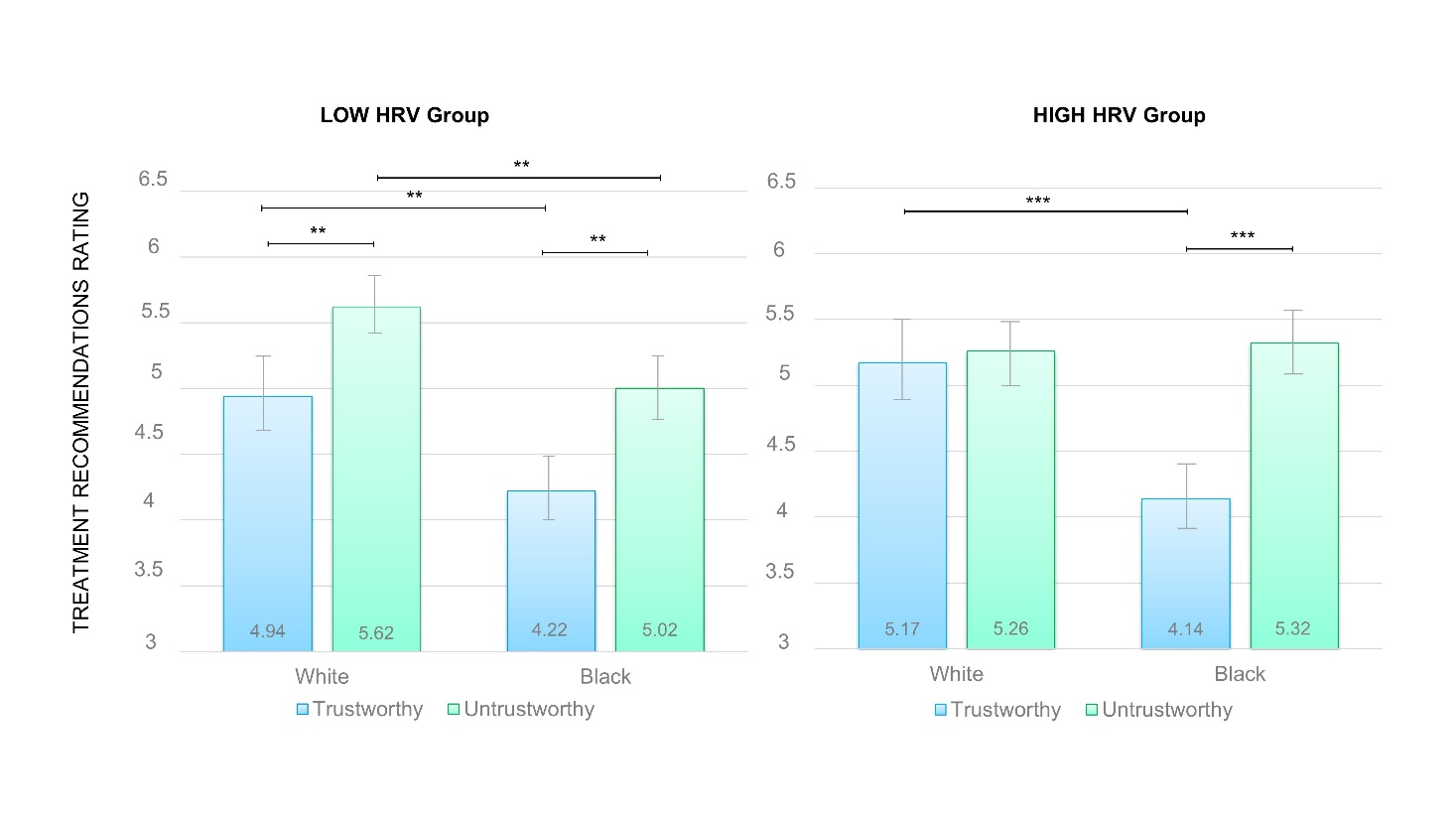
*Note***.** HRV = Heart Rate Variability; ****** *p* <.01; *** *p* <.001
